# The New Minimally Invasive Techniques for Pilonidal Sinus Disease Surgery: Choosing the Right Technique for the Right Patient—A Narrative Review

**DOI:** 10.3390/medicina62030518

**Published:** 2026-03-11

**Authors:** Matteo Matteucci, Lorenza Zampino, Gisella Barone, Andrea Carnevali, Luca Scaravilli, MiMi Yen, Salvatore Costeri, Camillo Leonardo Bertoglio

**Affiliations:** 1 Department of Medicine and General Surgery, University of Milan, 20122 Milan, Italy; mimi.yen@unimi.it; 2 Department of Surgery, Ospedale Giuseppe Fornaroli, ASST-Ovest Milanese, 20013 Magenta, Italy; lorenza.zampino@asst-ovestmi.it (L.Z.); gisella.barone@asst-ovestmi.it (G.B.); andrea.carnevali@asst-ovestmi.it (A.C.); luca.scaravilli@asst-ovestmi.it (L.S.); salvatore.costeri@asst-ovestmi.it (S.C.); camilloleonardo.bertoglio@asst-ovestmi.it (C.L.B.)

**Keywords:** pilonidal sinus disease, endoscopic pilonidal sinus treatment (EPSiT), off-midline closure, flap reconstruction, laser therapy, SiLac, surgical colorectal guidelines

## Abstract

*Background and Objectives*: Pilonidal sinus disease (PSD) is a common condition affecting predominantly young adults and it is associated with a substantial impact on quality of life. Despite the wide range of available surgical options, optimal management remains controversial due to heterogeneous recurrence rates, wound complications and prolonged recovery. Over the last decade, a paradigm shift has occurred from midline excision toward off-midline closure techniques and minimally invasive procedures. This narrative review aims to critically appraise contemporary surgical strategies for PSD, comparing outcomes, indications and limitations, with particular emphasis on patient selection. *Materials and Methods*: A narrative review of the literature was conducted using PubMed, Scopus, and major guideline repositories. Relevant randomized controlled trials, systematic reviews, meta-analyses, observational studies and national and international guidelines were reviewed and synthesized, with a focus on healing time, recurrence, postoperative complications and patient-reported outcomes. *Results*: Midline primary closure is consistently associated with higher recurrence and complication rates and is no longer recommended. Off-midline flap techniques provide the most durable results in recurrent or complex disease. Minimally invasive approaches, including endoscopic and laser-assisted techniques, offer faster recovery and high short-term patient satisfaction in selected primary cases, but should not be considered equivalent to flap surgery in complex or recurrent PSD due to limited long-term evidence. Current guidelines uniformly advocate individualized, patient-tailored treatment strategies and discourage midline closure. *Conclusions*: Surgical management of PSD should be individualized according to disease complexity, prior interventions and patient expectations. While minimally invasive techniques represent an attractive option for selected primary disease, off-midline flap procedures remain the standard of care for extensive or recurrent cases. High-quality comparative studies with standardized outcome definitions and long-term follow-up are required to refine treatment algorithms.

## 1. Introduction

Pilonidal sinus disease (PSD) is a chronic inflammatory condition of the sacrococcygeal region, characterized by sinus tracts containing hair and debris within the natal cleft. First described by Hodges in 1880 [1], the term *pilonidal* reflects the etiological role of hair penetration into subcutaneous tissue. PSD predominantly affects young adults, with a marked male predominance, and represents a frequent cause of surgical referral in general surgery practice. Although the epidemiology and predisposing factors of PSD, such as deep natal cleft anatomy, obesity, local maceration, and hirsutism [2,3,4,5], are well established, its optimal surgical management remains controversial. The disease exhibits a broad clinical spectrum [6,7,8], ranging from asymptomatic midline pits to recurrent abscesses and complex, branching sinus systems. This heterogeneity, combined with a tendency toward recurrence and wound complications, has resulted in the development of numerous surgical techniques over the past century. Historically, wide excision with midline closure or healing by secondary intention represented the standard approach. However, these techniques are associated with prolonged healing times, wound morbidity and unacceptably high recurrence rates [9,10,11]. In an attempt to improve wound healing and reduce postoperative complications, adjunctive strategies such as negative pressure wound therapy (NPWT) have also been introduced, particularly in cases managed with open healing or after wide excision. NPWT has been proposed to promote granulation tissue formation, reduce wound exudate, and potentially shorten healing time, although evidence remains heterogeneous and largely based on small series [12]. As a result, contemporary management has progressively shifted toward off-midline closure techniques and, more recently, toward minimally invasive procedures aimed at reducing surgical trauma and accelerating recovery. Despite the growing popularity of endoscopic, laser-assisted, and other minimally invasive techniques, uncertainty persists regarding their long-term effectiveness, optimal indications, and equivalence to established flap-based procedures, particularly in recurrent or complex disease. Moreover, existing guidelines [13,14,15,16] consistently discourage midline closure but differ in their recommendations for newer techniques, largely due to the paucity of high-quality comparative data. This narrative review aims to support clinical decision-making by clarifying the “right technique for the right patient” in pilonidal sinus disease, rather than conducting a formal systematic review or meta-analysis of comparative outcomes.

## 2. Materials and Methods

This study was conducted as a narrative review of the contemporary literature on the surgical management of pilonidal sinus disease.

### 2.1. Search Strategy and Selection Criteria

A comprehensive literature search was performed using PubMed, Scopus and Web of Science databases by two authors (M.M. and L.Z.) from January 2025. The final literature search was completed in June 2025.

The search strategy included combinations of the following keywords: pilonidal sinus disease OR PSD AND off-midline closure AND flap reconstruction AND endoscopic pilonidal sinus treatment OR EPSiT AND video-assisted ablation of pilonidal sinus OR VAAPS AND laser therapy OR SiLaC AND minimally invasive surgery OR minimally invasive techniques. Additional strategies included reviewing the references of all included articles for further relevant material.

Studies were selected according to predefined eligibility criteria to ensure clinical relevance and methodological consistency. We included original clinical investigations evaluating surgical treatment for PSD in adult patients (≥18 years). Eligible studies included randomized controlled trials, non-randomized comparative studies, prospective cohort studies, and retrospective clinical series enrolling at least 10 patients per treatment arm. To ensure meaningful clinical interpretation, studies were required to report at least one clearly defined outcome measure, such as recurrence rate, time to complete wound healing, surgical site infection, time to return to normal activities or work, or validated patient-reported outcomes. When recurrence was assessed, a minimum follow-up of six months was considered necessary for inclusion. In addition, international guidelines and consensus statements from recognized scientific societies were included to contextualize current standards of practice. Only full-text articles published in peer-reviewed journals in English were considered.

Studies were excluded if they consisted solely of case reports, very small case series (fewer than 10 patients), narrative reviews, systematic reviews, editorials, expert opinions, letters to the editor, or conference abstracts without full-text publication. Studies focusing exclusively on pediatric populations or failing to provide extractable clinical outcome data were also excluded. In cases of overlapping patient cohorts, the most comprehensive or most recent publication was retained to avoid duplication of data.

**Figure 1:**
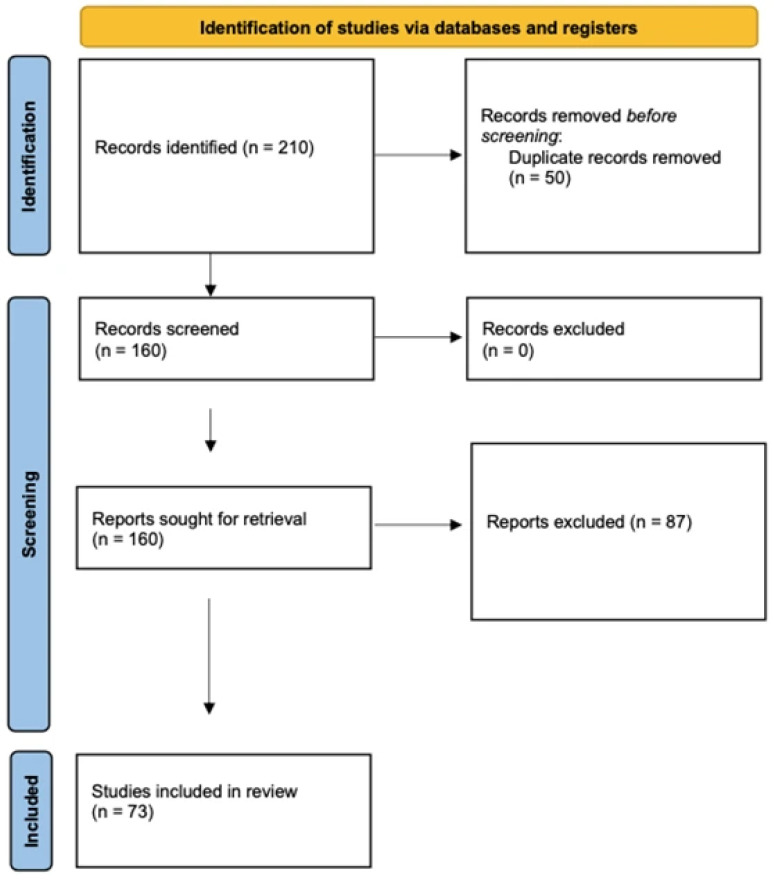
Study selection process. Flow diagram illustrating the identification, screening, eligibility assessment, and inclusion of studies in this narrative review, structured according to the principles of the PRISMA Statement. The diagram reports the number of records identified through database searching, duplicates removed, titles and abstracts screened, full-text articles assessed for eligibility, and studies included in the qualitative synthesis, together with the main reasons for exclusion at the full-text stage.

The literature search identified approximately 210 records. After removal of duplicates, 160 titles and abstracts were screened independently by two reviewers. Full texts of 160 potentially eligible studies were assessed for eligibility. Discrepancies were resolved through discussion and, when necessary, consultation with a third reviewer (G.B.). Ultimately, a total of 73 studies met the inclusion criteria and were included in the qualitative synthesis. The study selection process was conducted in accordance with the principles outlined in the PRISMA Statement (Figure 1).

The literature was synthesized qualitatively, with particular attention to clinically relevant endpoints, including healing time, recurrence rates, postoperative complications, patient-reported outcomes and return to normal activities. Given the heterogeneity of study designs, outcome definitions and follow-up durations, no formal statistical pooling or meta-analysis was performed. Instead, evidence was critically appraised to highlight comparative advantages, limitations, and appropriate indications for each surgical approach.

### 2.2. Data Extraction

All included studies were reviewed by two independent reviewers (M.M and L.Z.). For each study, the following variables were recorded: study design, sample size, patient demographics, disease characteristics (primary vs. recurrent, severity when available), type of surgical technique, adjunctive treatments, follow-up duration, and reported outcomes. Any discrepancies in study selection or data interpretation were resolved through discussion and consensus

### 2.3. Study Outcomes

The primary outcomes of interest were healing time, recurrence, intraoperative and post-operative complications and patient satisfaction. To enhance comparability across studies, outcomes were interpreted using standardized operational definitions whenever possible.

Recurrence was defined as any of the following occurring after complete wound healing: need for re-intervention (surgical or minimally invasive), clinical reappearance of a symptomatic sinus or midline pit, or imaging-confirmed recurrent tract.

When data allowed, recurrence rates were interpreted according to predefined follow-up intervals (12, 24, and 60 months) to reduce heterogeneity related to variable definitions of “short-term” and “long-term” outcomes across studies.

Healing was considered complete epithelialization of the surgical wound or complete closure of the treated cavity, depending on the surgical technique employed. When studies provided alternative definitions, these were interpreted considering this operational framework to facilitate meaningful comparison.

Patient satisfaction included time to return to work or school and postoperative analgesic requirements, as these parameters are highly relevant in a predominantly young and working population affected by pilonidal sinus disease.

## 3. Traditional Management Approaches: A Historical Perspective

The surgical management of PSD has evolved significantly since its first description in the 19th century. The earliest surgical strategies consisted of wide excision of the sinus tract and surrounding tissue, with secondary intention healing. This approach, historically preferred for minimizing infection, was associated with prolonged healing times, up to 8–12 weeks, and significant patient discomfort [17,18,19]. To accelerate recovery, midline primary closure was introduced in the early 20th century [18,19,20]. Although it shortened healing time, long-term outcomes revealed unacceptably high recurrence rates, ranging from 20% to 30%, and elevated wound complication rates, including dehiscence and surgical site infection [18,19,20]. The inherent disadvantage lies in the placement of the suture line within the deep natal cleft, a region predisposed to moisture, maceration and local tension. Several modifications aimed to improve these results, including marsupialization [21,22,23], which involved suturing wound edges to the fascia to promote granulation and reduce dead space. While marsupialization decreased recurrence relative to wide excision, it still required extended wound care and delayed return to normal activity, limiting its practicality [21,22,23].

## 4. Recent Surgical Innovations

Recent innovations primarily involve minimally invasive strategies, advanced flap procedures, laser-assisted methods and a universal trend towards off-midline closure.

### 4.1. Endoscopic Pilonidal Sinus Treatment, Pit-Picking and Sinusectomy

**Figure 2:**
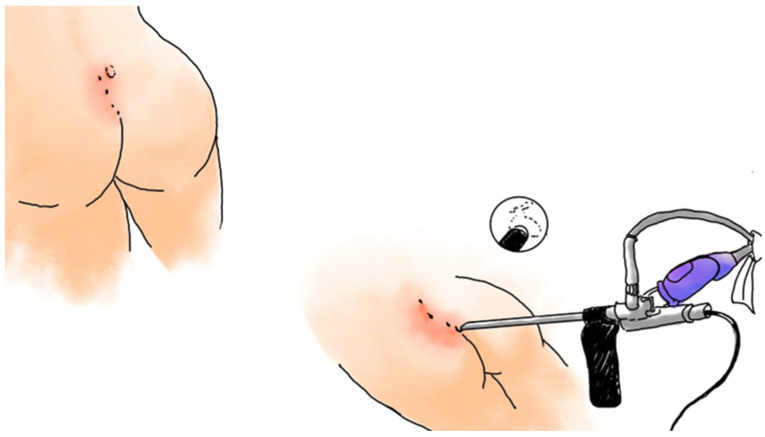
Endoscopic Pilonidal Sinus Treatment (EPSiT). The procedure employs a rigid fistuloscope introduced through the sinus opening, allowing direct visualization of the sinus tract and its lateral branches. Hair, debris, and granulation tissue are removed using endoscopic forceps, and the epithelial lining is ablated with a monopolar electrode under continuous irrigation, preserving surrounding healthy tissue and promoting rapid healing.

One of the most significant breakthroughs in PSD surgery has been the introduction of endoscopic-assisted approaches, particularly Endoscopic Pilonidal Sinus Treatment (EPSiT), first described by Meinero in 2014 [24]. The procedure is typically performed under local or spinal anesthesia with the patient in the prone jackknife position. After introducing the fistuloscope through the external opening, the surgeon systematically explores all sinus tracts under direct vision. Hair nests, debris, and granulation tissue are meticulously removed using endoscopic forceps or a brush, while the epithelial lining is ablated using a monopolar electrode or laser fiber inserted through the operative channel. Continuous irrigation ensures a clear visual field and facilitates thorough debridement. Complete clearance of hair and debris under direct visualization is critical to optimize outcomes. Once all tracts are cleaned and coagulated, the cavity is left open to heal by secondary intention, without the need for sutures or drainage (Figure 2). Patient selection is crucial for optimal results. EPSiT and other endoscopic approaches are best suited for patients with limited midline pits, minimal lateral branching, and no extensive sepsis or off-midline secondary openings. Favorable outcomes are closely linked to appropriate case selection, meticulous technique, and adequate equipment availability. Moreover, these procedures require a structured learning curve, which may influence early recurrence rates in less experienced hands. EPSiT is associated with shorter hospital stays, minimal postoperative pain, and early return to normal activities, with average healing times of approximately three to four weeks [24,25,26,27]. Recurrence rates are generally reported between 5% and 10% at one to three years in selected uncomplicated cases [24,25,26,27]. However, limited long-term data beyond this timeframe and the need for specialized equipment remain barriers to universal adoption [28]. Similarly, pit-picking [29,30] and sinusectomy [31] represent alternative minimally invasive options, typically performed under local anesthesia in outpatient settings. These techniques offer very short recovery times, often less than two weeks, but favorable outcomes tend to cluster in patients with simple primary disease, shallow natal clefts, and limited tract extension. Failures frequently reflect unrecognized lateral tracts; therefore, preoperative ultrasound may be useful in patients with equivocal anatomy to better delineate sinus complexity. Long-term recurrence rates may reach 15–40%, particularly in complex or recurrent disease [29,30,31]. Consequently, pit-picking and limited sinusectomy should be reserved for carefully selected early-stage PSD with simple anatomical features.

### 4.2. Flap-Based Procedures

**Figure 3:**
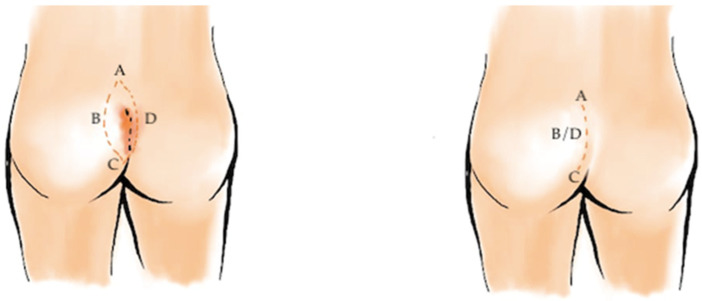
Karydakis flap procedure for pilonidal sinus disease. An asymmetric elliptical excision of the sinus and surrounding tissue is performed, followed by mobilization of a lateral fasciocutaneous flap. The flap is advanced medially to cover the defect, displacing the suture line off the midline, reducing tension and minimizing recurrence risk. Red lines delineate the planned asymmetric elliptical excision and the lateral limits of flap mobilization, while reference letters (A, B, C and D) identify key anatomical landmarks and the direction of flap advancement.

**Figure 4:**
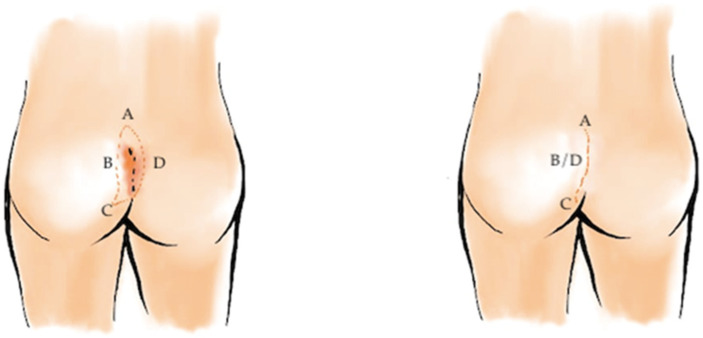
Bascom cleft-lift procedure for pilonidal sinus disease. The midline pits and fibrotic tracts are excised while preserving surrounding healthy tissue. A laterally based skin flap is mobilized and secured to the presacral fascia, effectively flattening and lateralizing the intergluteal cleft to reduce tension and lower recurrence risk. Red lines delineate the planned asymmetric elliptical excision and the lateral limits of flap mobilization, while reference letters (A, B, C and D) identify key anatomical landmarks and the direction of flap advancement.

Despite the appeal of minimally invasive surgery, flap-based techniques remain the cornerstone of treatment for extensive or recurrent disease. These procedures share a common principle: flattening the natal cleft and relocating the incision away from the midline, thereby reducing hair accumulation and local moisture, two key factors in recurrence. Among these, the Karydakis flap (Figure 3) and the Bascom cleft-lift (Figure 4) have become widely accepted for their reproducibility and favorable outcomes. Healing typically occurs within two to three weeks, and recurrence rates range from 1% to 4%, substantially lower than with traditional methods [32,33,34,35,36,37,38,39,40,41,42].

The Karydakis procedure, first described in the 1970s [32], involves elliptical asymmetric excision of the sinus complex with its tract and a small margin of surrounding tissue [16]. A large fasciocutaneous flap is then mobilized from the lateral gluteal region and advanced medially to cover the defect, displacing the suture line off the midline. The subcutaneous layer is usually closed with absorbable sutures, and a suction drain may be placed to prevent seroma formation (Figure 2).

The Bascom cleft-lift, a modification of the Karydakis concept, utilizes a more limited excision, focusing on removing only the midline pits and fibrotic tracts while preserving most of the surrounding healthy tissue. A laterally based skin flap is elevated and secured to the presacral fascia, effectively shallowing the intergluteal cleft. This approach minimizes tissue tension and allows for rapid wound healing with excellent cosmetic and functional outcomes [43].

**Figure 5:**
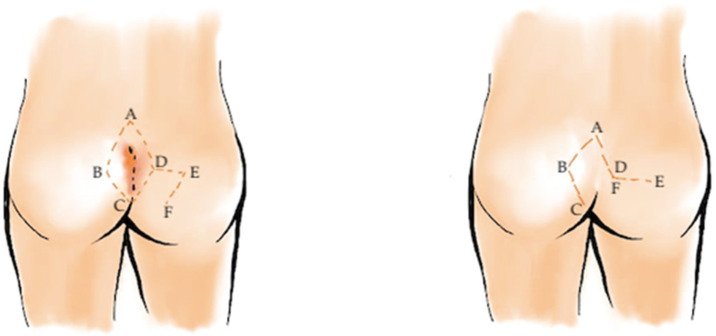
Limberg (rhomboid) flap procedure for pilonidal sinus disease. A rhomboid-shaped excision of the sinus and surrounding tissue is performed, and a fasciocutaneous flap of identical dimensions is transposed obliquely to cover the defect. The lateralized closure off the midline reduces tension, promotes healing, and minimizes recurrence risk. Red lines delineate the planned asymmetric elliptical excision and the lateral limits of flap mobilization, while reference letters (A, B, C, D, E and F) identify key anatomical landmarks and the direction of flap advancement.

The Limberg (rhomboid) flap (Figure 5) offers another reliable solution, particularly for complex sinuses [18,19,20]. The procedure involves a rhomboid-shaped excision of all diseased tissue, typically encompassing the sinus openings and underlying tracts, with the defect’s sides measuring equal lengths and angles of 60° and 120°. A fasciocutaneous flap of identical dimensions is then designed from one gluteal side and transposed obliquely to cover the defect. The donor area is closed primarily, creating a lateralized scar that avoids the midline and reduces the risk of recurrence. Its geometric design allows both wide excision and tension-free closure, with recurrence rates generally below 5% [44]. Recent modifications of the Limberg flap have further improved long-term results, reducing recurrence to 4.9% in five years compared to 7.1% for the classical technique [32,33,34,35,36,37,38,39,40,41,42,43,44]. These refinements underscore the continuous evolution of flap surgery toward greater durability and patient comfort.

When selecting an off-midline flap, practical anatomical and disease-related considerations can guide decision-making. The Karydakis flap is widely regarded as a reproducible and technically straightforward procedure, characterized by lateralization of the suture line and flattening of the natal cleft. Its relative simplicity, broad availability, and consistent outcomes make it a reliable option for both primary and recurrent disease of moderate extent. The Bascom cleft-lift procedure may be particularly advantageous in patients with deep or narrow clefts and in those with prior midline scarring, as it focuses on cleft shallowing and off-midline closure while minimizing wide tissue mobilization. In contrast, the Limberg flap and its modifications are especially useful in patients with broad disease fields, multiple lateral tracts, or extensive recurrent PSD, as the rhomboid excision allows generous removal of affected tissue and tension-free reconstruction. This broader excision, however, may come at the cost of a longer incision and potentially more extensive tissue mobilization. Thus, flap selection should be individualized based on cleft morphology, disease extent, previous surgical history, and surgeon expertise. Beyond flap design, several modifiable technical factors influence postoperative outcomes. The use of closed-suction drains after off-midline flap surgery has been variably associated with reduced fluid collections, although evidence on their impact on wound healing and infection is mixed; in randomized and comparative studies drains decreased fluid collections but did not consistently translate into improved clinical endpoints such as infection, recurrence, or faster healing, and their use often depends on surgeon preference and individual patient factors. A prospective randomized trial of Karydakis flap with compressing interrupted sutures and no drain demonstrated lower seroma rates and fewer wound complications compared with standard Karydakis with drain, suggesting that suture techniques and dead-space control may mitigate the need for drains in selected cases [45]. NPWT applied over primary off-midline closures such as Bascom cleft-lift has been studied in randomized settings; recent RCTs did not show significant benefit in wound healing rates or reduction in complications compared to conventional dressings or short-duration drains after such procedures [12,46]. Meticulous control of dead space through layered closure, quilting or progressive tension sutures, and careful hemostasis are critical in minimizing seroma and wound dehiscence, principles supported broadly in surgical wound management literature and extrapolated to PSD flap surgery. Although high-quality comparative data on quilting and progressive tension sutures specifically in PSD are limited, analogous evidence in soft tissue procedures underscores their potential value in seroma prevention and tension reduction. Overall, careful surgical technique and thoughtful postoperative wound care protocols likely play a more substantial role in optimizing flap durability and reducing complications than reliance on any single adjunctive measure.

### 4.3. Laser-Assisted Methods

**Figure 6:**
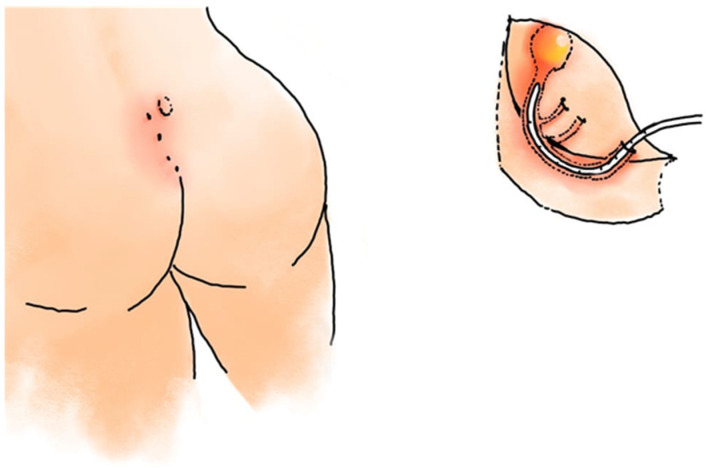
Schematic representation of Sinus Laser Closure (SiLaC) for pilonidal sinus disease. The procedure involves insertion of a radial-emitting laser fiber into the sinus tract, followed by controlled laser energy delivery to ablate the epithelial lining and coagulate the surrounding tissue, promoting tract closure while preserving healthy surrounding tissue. Continuous irrigation ensures debris removal and tract visualization throughout the procedure.

Laser technology has recently been introduced as an adjunctive or primary modality for PSD treatment. The Sinus Laser Closure (SiLaC) technique employs a diode laser delivered through a radial fiber to thermally obliterate the sinus tract [47]. The procedure is typically performed under local or spinal anesthesia in the prone position (Figure 6). After identifying and gently dilating the sinus opening, the tract is carefully curetted and irrigated to remove hair and debris. A 1470 nm or 980 nm diode laser fiber (usually 360° radial emission, 400–600 μm in diameter) is then introduced through the external orifice and slowly withdrawn under continuous laser activation. The delivered energy (usually 10–12 W, total 400–600 J per tract) induces coagulative necrosis and shrinkage of the sinus walls, promoting secondary closure by fibrosis. The procedure preserves the overlying skin and surrounding subcutaneous tissue, minimizing postoperative pain and scarring. Early clinical studies report encouraging results, with healing typically achieved within two to four weeks and recurrence rates between 4% and 8% at short-term follow-up [48,49,50,51,52,53]. Laser therapy offers excellent cosmetic outcomes and can often be performed in an outpatient setting under local anesthesia. However, like EPSiT, the requirement for specialized devices and the absence of robust long-term data limits its widespread use [48,49,50,51,52,53].

### 4.4. Radiofrequency-Assisted Excision

Radiofrequency (RF)–based techniques have emerged as minimally invasive alternatives for the management of PSD, but the term encompasses heterogeneous approaches. Some authors describe RF ablation–only techniques aimed at destroying the sinus epithelium in situ, whereas others report RF-assisted excision, in which high-frequency alternating current is used to perform a limited excision with simultaneous coagulation [54]. This distinction is relevant, as indications, wound management, and outcomes may differ between purely ablative strategies and RF-guided excisional procedures. RF technology induces localized heating that results in controlled tissue coagulation, epithelial ablation, and effective hemostasis, thereby reducing intraoperative bleeding and potentially limiting postoperative inflammation. The procedure is performed with the patient in a prone or jackknife position under local, regional, or short general anesthesia, depending on disease extent and institutional protocols. Comparative studies, such as the pilot randomized trial by Gupta et al. (2005) [55], have reported shorter operative times (approximately 10 min vs. 36 min), reduced hospital stays (median ~9 h vs. ~30 h), fewer analgesic requirements, and earlier return to work (~6 days vs. ~16 days) for RF-assisted excision compared with conventional excision with marsupialization. Reported mean wound-healing times were also shorter in the RF group (approximately 49 days vs. 84 days). Recurrence rates in early series have appeared low (e.g., one recurrence per arm at two-year follow-up in the Gupta trial), although follow-up durations have generally been limited (median ~30 months) [55,56]. Importantly, most available studies compare RF techniques with open excision rather than with off-midline flap procedures, which are currently considered standard for more complex disease. This limits the strength of inferences that can be drawn regarding the role of RF in advanced or recurrent PSD. Moreover, the literature is characterized by small sample sizes, heterogeneous follow-up intervals, and a lack of large-scale randomized trials. Consequently, while RF-based approaches appear promising in selected patients, particularly those with limited disease, their long-term durability and comparative effectiveness in complex PSD remain to be clearly established [55,56].

### 4.5. Platelet-Rich Plasma Applications in PSD Treatment

In recent years, the adjunctive use of autologous platelet-rich plasma (PRP) has emerged as a promising strategy in the surgical management of PSD [57]. PRP is obtained from the patient’s own whole blood by a process of centrifugation that separates platelet-rich plasma from platelet-poor plasma and red blood cells. The concentrated platelet fraction is then activated (for example, by calcium gluconate or thrombin) to release a rich milieu of growth factors and cytokines, such as platelet-derived growth factor (PDGF), transforming growth factor-β1 (TGF-β1), vascular endothelial growth factor (VEGF), fibroblast growth factor-2 (FGF-2), and insulin-like growth factor (IGF-1), which collectively promote angiogenesis, fibroblast proliferation, collagen synthesis and epithelisation of wounds. From a technical perspective, the application of PRP in PSD surgery is most often performed after excision or curettage of the offending sinus tracts and cavities. Techniques vary, but a representative protocol is as follows: once the sinus or cavity has been debrided, the wound cavity volume is measured (for example by saline infusion technique), and the PRP gel (or liquid) is instilled to fill the cavity completely, sometimes followed by placement of a PRP-impregnated dressing or injection into the wound edges. For instance, in a randomized controlled trial by Gohar et al. [58], patients undergoing the lay-open technique for PSD received PRP injections into the wound on postoperative days 4 and 12 and achieved a mean healing time of 45 ± 2.6 days compared with 57 ± 2.4 days in the control group (*p* = 0.001). In another RCT by Boztug et al. [59], open surgery followed by PRP application achieved significantly faster recovery per unit cavity volume and lower pain scores compared to open surgery alone (with 22 patients in the PRP group). A further systematic review by Khan et al. (2022) [60] specifically targeting healing time found a mean difference of −13.01 days (95% CI 12.15–13.86; *p* < 0.00001) for PRP vs. control. Nevertheless, PRP should be regarded as an adjunctive measure aimed primarily at enhancing and accelerating epithelialization and wound maturation rather than as a stand-alone treatment or a substitute for definitive surgical management [61,62,63]. Substantial heterogeneity exists among studies with respect to PRP preparation systems, platelet concentration achieved, activation methods, injected volume relative to cavity size, and timing of application (intraoperative versus delayed postoperative administration). This variability limits comparability and may partially explain differences in reported outcomes. Future studies should clearly report platelet concentration (e.g., fold increase over baseline), total injected volume, method of activation, and precise timing and frequency of application to reduce methodological heterogeneity and enable meaningful comparisons across trials. Moreover, evidence regarding long-term recurrence prevention remains scarce, and extended follow-up is needed before firm conclusions can be drawn, particularly in complex or recurrent PSD.

## 5. Comparative Outcomes

Recent systematic reviews and meta-analyses published between 2018 and 2024 [61,62,63,64,65,66] have clarified the relative performance of the main surgical strategies for PSD. However, comparisons must be interpreted considering three critical variables: (1) disease severity (primary limited vs. complex/recurrent), (2) duration of follow-up, and (3) study design. Off-midline flap procedures are supported by long-term randomized and meta-analytic data, whereas minimally invasive and energy-based techniques are predominantly evaluated in selected primary disease with shorter follow-up, limiting the strength of durability conclusions. The outcomes most consistently reported across reviews include healing time, recurrence, complications, and patient satisfaction.

In the large meta-analysis by Stauffer et al. (Table 1) [61], recurrence rates varied markedly according to surgical technique and length of follow-up. Primary midline closure demonstrated progressively increasing recurrence over time, reaching 21.9% at 60 months in RCTs and up to 67.9% at 240 months in non-RCTs. In contrast, off-midline procedures such as the Karydakis and Bascom cleft lift techniques maintained substantially lower long-term recurrence (≤1.9% at 60 months in non-RCTs), while Limberg flap techniques showed low early recurrence (0.4% at 12 months in RCTs) with moderate increases at extended follow-up. Marsupialization and pit-picking were associated with acceptable short-term outcomes but demonstrated rising recurrence at 60 months (9.4% and 15.6%, respectively). SiLaC showed low early recurrence (1.9% at 12 months), but recurrence increased to 36.6% at 60 months.

In the Cochrane review by Al-Khamis et al. (Table 2) [64], primary midline closure was associated with significantly longer healing time compared with other closed techniques (MD 5.40, 95% CI 2.28–8.52), higher surgical site infection (SSI) rates (RR 3.72, 95% CI 1.86–7.42), and increased recurrence beyond 12 months (Peto OR 5.76, 95% CI 2.53–13.11). Bascom techniques showed variable healing times (MD 9.9), whereas Limberg flap demonstrated shorter healing in comparative analyses (MD 2.40). Return-to-work data were inconsistently reported.

The systematic review by Emile et al. (Table 3) [62] evaluating EPSiT, primarily used for chronic, limited sacrococcygeal sinus tracts and frequently performed under local anesthesia with or without sedation, reported a low failure/recurrence rate of 6.3%, minimal SSI (1.1%), rapid return to work (2.9 ± 1.8 days), and high patient satisfaction (95.6%).

In the comparative meta-analysis by Enriquez-Navascués et al. (Table 4) [63], midline closure was associated with higher infection risk compared to off-midline primary closure or flap procedures (RR 2.75, 95% CI 1.83–4.13), while recurrence differences at 12 months did not reach statistical significance (RR 2.32, 95% CI 0.98–5.45). When comparing Karydakis/Bascom versus Limberg techniques, no significant difference in recurrence was observed (RR 1.12), although return to work slightly favored Karydakis/Bascom (MD −0.18 days).

Regarding laser therapy, Qin et al. (Table 5) [65] reported for SiLaC an overall healing rate of 81.9%, with 74.5% healing beyond 12 months follow-up, delayed healing in 11.1%, and mild postoperative pain in 18.5% of cases. However, long-term recurrence concerns are highlighted in the Stauffer analysis.

Finally, in the meta-analysis by Wiinblad et al. (Table 6) [66], flap techniques demonstrated significantly lower recurrence and infection compared to primary closure (OR 0.31 and OR 0.33, respectively). Secondary intention healing showed comparable recurrence to Limberg techniques (OR 0.38) but lower infection rates (OR 0.48). No significant difference in recurrence was found between Karydakis and Limberg procedures, although infection rates were higher with Karydakis (OR 2.05).

Overall, across the referenced studies, off-midline flap techniques (Karydakis, Bascom, Limberg), typically performed under spinal or general anesthesia for chronic or complex disease, consistently demonstrate superior long-term recurrence control and lower infection rates compared with primary midline closure. Minimally invasive techniques such as EPSiT and SiLaC, often performed under local anesthesia and mainly applied to limited primary sinuses, provide faster return to work and higher patient satisfaction, although concerns remain regarding long-term recurrence with laser-based approaches.

To ensure appropriate interpretation of the data summarized in Table 1, Table 2, Table 3, Table 4, Table 5 and Table 6, outcomes should be analyzed considering both follow-up duration and underlying study design. Recurrence is a time-dependent outcome, and short-term results (≤12 months) primarily reflect early technical success rather than true long-term disease control. As illustrated in Table 1 (Stauffer et al. [61]), recurrence rates after primary midline closure increase progressively over extended follow-up, reaching markedly higher values at 60–240 months compared with early assessments. In contrast, off-midline flap procedures such as Karydakis, Bascom cleft lift, and Limberg demonstrate relatively stable recurrence curves over time, especially in studies with longer follow-up. Similarly, the comparative findings summarized in Table 2, Table 4 and Table 6, derived from meta-analyses including randomized controlled trials, consistently show higher infection risk and less favorable long-term recurrence profiles for midline closure compared with off-midline techniques. Importantly, some comparisons do not demonstrate statistically significant recurrence differences at 12 months, highlighting how short-term analyses may underestimate divergence between techniques that becomes evident only with prolonged follow-up. By contrast, the evidence summarized in Table 3 and Table 5 for minimally invasive approaches such as EPSiT and SiLaC is predominantly based on prospective series and non-randomized studies, often conducted in selected patients with limited primary disease and with shorter observation periods. These techniques consistently show rapid return to work, low early morbidity, and high patient satisfaction. However, as suggested by longer-term pooled analyses reported in Table 1, recurrence rates for certain energy-based techniques may increase over time, underscoring the need for cautious interpretation when long-term randomized data are lacking. Taken together, the data presented across the tables support a differentiated interpretation: off-midline flap procedures are supported by higher-level comparative evidence with extended follow-up, strengthening conclusions regarding durable recurrence control. Minimally invasive and energy-based techniques demonstrate clear short-term advantages but are currently supported by more limited long-term evidence. Therefore, when proposing treatment positioning, distinctions are made according to both the strength of evidence and the time horizon of reported outcomes, to avoid conflating early postoperative benefits with long-term disease durability.

## 6. National and International Guidelines

National and international guidelines consistently converge on several key principles in the surgical management of pilonidal sinus disease (PSD). Across documents from the Italian Society of Colorectal Surgery (SICCR), the American Society of Colon and Rectal Surgeons (ASCRS), the German S3 guideline group, and the European Society of Coloproctology (ESCP), there is uniform discouragement of traditional midline primary closure due to its higher risk of wound complications and recurrence.

♦ **Strong against:** Midline primary closure.
Both the ASCRS [13] and German S3 guideline [15], in line with earlier SICCR recommendations [14], clearly discourage midline closure, citing consistent evidence of inferior outcomes compared with off-midline techniques.
♦ **Strong for:** Off-midline flap reconstruction in complex or recurrent disease.
All major guidelines endorse off-midline closure techniques—such as Karydakis, Bascom cleft-lift, or Limberg-type flaps—for extensive, recurrent, or anatomically complex disease. These approaches are considered the most reliable option for durable disease control when significant lateral extension, multiple tracts, or prior surgical failure are present.
♦ **Conditional for:** Minimally invasive techniques in selected primary disease.
Minimally invasive approaches (e.g., sinusectomy, pit-picking, EPSiT/VAAPS) are conditionally supported for limited, uncomplicated primary PSD. The SICCR and German S3 guidelines explicitly recommend these techniques in carefully selected patients with simple anatomy, while the ESCP highlights their advantages in terms of postoperative pain and early return to daily activities, albeit with recognition of limited long-term recurrence data [16].

While emerging technologies such as laser ablation (SiLaC) and radiofrequency-assisted procedures are acknowledged in contemporary literature, guideline endorsement remains cautious. Recommendations are generally conditional or absent due to limited high-quality randomized evidence, heterogeneous protocols, and insufficient long-term follow-up. As such, these modalities are best considered promising adjuncts or alternative options in selected cases rather than established standards of care.

Overall, major guidelines emphasize individualized, patient-centered decision-making that integrates disease complexity, previous surgical history, anatomical features, and surgeon expertise. They also uniformly call for standardized outcome definitions and high-quality randomized trials to strengthen future recommendations. Table 7 summarizes surgical strategies for PSD according to SICCR, ASCRS, ESCP, and the German S3 guideline.

## 7. Controversies and Current Limit

Despite advances in the surgical management of PSD, controversies persist due to the complexity of the disease, the variety of surgical techniques, and limited high-quality evidence. A major limitation is the scarcity of large, multicenter randomized controlled trials (RCTs). Most studies are single-center case series, retrospective analyses, or small prospective cohorts, reducing the ability of standardization and writing of universally valid guidelines. Meta-analyses and systematic reviews attempt to pool data, but heterogeneity in patient selection, disease severity, and outcome definitions complicates interpretation. For instance, recurrence is variably defined, from clinical finding of sinus tracts to patient-reported symptoms, resulting in inconsistent reporting. Surgical approaches such as midline excision, secondary intention healing, off-midline flaps, minimally invasive endoscopic methods and laser-assisted techniques are extremely variables in different studies and centers making comparisons difficult. Each technique differs in learning curve, resources, and postoperative care. Patient-specific factors, including sinus complexity, body habitus, and prior interventions, also influence outcomes but are inconsistently reported. Considerations of equity, resource availability, and health-system organization are essential when translating evidence into practice for PSD. Although endoscopic, laser, and radiofrequency-based techniques offer attractive short-term benefits, they are inherently equipment-dependent and may be unrealistic in low-resource settings where dedicated scopes, laser generators, disposable fibers, or maintenance infrastructure are unavailable or not reimbursed. In such contexts, pragmatic alternatives, such as sinusectomy with meticulous tract probing, limited excision with careful lateral exploration, or standardized off-midline flap procedures, represent acceptable and effective strategies. A well-executed sinusectomy performed under local anesthesia, with thorough identification of secondary tracts, may serve as a reasonable bridge approach when advanced technologies are inaccessible, without compromising fundamental surgical principles. Conversely, minimally invasive and off-midline procedures lend themselves well to structured day-surgery pathways and local or regional anesthesia protocols, potentially reducing hospital stay, indirect societal costs, and time away from work. From an administrative and patient-centered perspective, cost components extend beyond operative time and include device acquisition, disposable materials (e.g., endoscopic instruments, laser fibers), need for repeat interventions, wound-care requirements, and productivity loss. Therefore, technique selection should consider not only anatomical and disease-related factors but also local expertise, infrastructure, reimbursement models, and broader economic implications. Framing PSD management within a resource-sensitive framework enhances generalizability and supports equitable access to effective care across diverse healthcare systems.

## 8. When Should Minimally Invasive Techniques Be Chosen?

Minimally invasive techniques are best suited for selected patients with limited primary disease, in whom the anatomical and clinical characteristics favor tract ablation rather than wide excision.

Ideal candidates typically include patients presenting with:

• primary PSD without prior surgical treatment.
• a limited number of midlines pits.
• short, non-branching sinus tracts.
• absence of extensive lateral extensions or complex cavities.
• no active acute abscess at the time of surgery.

**Figure 7:**
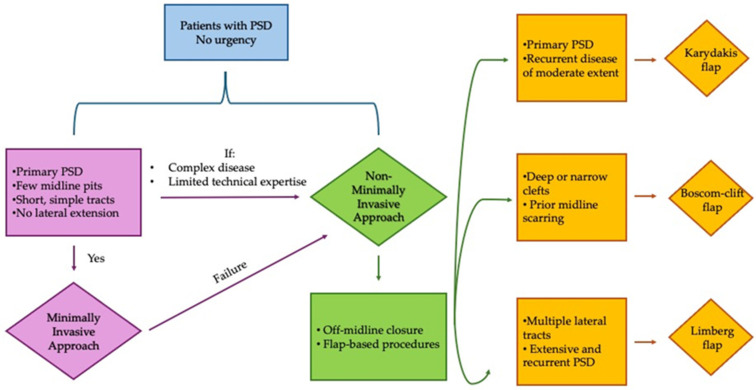
Proposed therapeutic decision-making algorithm for pilonidal sinus disease (PSD). The flowchart integrates disease severity (primary vs. recurrent/complex), sinus anatomy (limited vs. branching tracts), patient-related factors, and resource availability to guide the selection between minimally invasive approaches and off-midline flap procedures.

In this setting, techniques such as endoscopic treatment (EPSiT/VAAPS), pit-picking, or laser-assisted ablation can achieve rapid recovery, minimal postoperative pain, and high patient satisfaction, with acceptable short-term recurrence rates. These advantages are particularly relevant for young, working patients who prioritize early return to daily activities and favorable cosmetic outcomes. However, the indications for endoscopic techniques are evolving. Several groups have reported the use of PEPSiT and related endoscopic approaches even in patients with severe, recurrent, or anatomically complex disease, supported by dedicated severity classifications and standardized operative protocols [67]. These experiences suggest that, in selected cases and in experienced hands, endoscopic management may extend beyond simple primary PSD. Nevertheless, caution remains warranted. Previous surgery, multiple lateral tracts, deep or wide natal clefts, and extensive chronic inflammation are consistently associated with higher failure and recurrence rates when minimally invasive methods are employed. In such scenarios, intraluminal ablation alone may fail to adequately address both the underlying disease burden and the predisposing anatomical factors. Importantly, although short-term patient-reported outcomes after minimally invasive procedures may appear comparable to those achieved with flap-based surgery, these techniques should not be considered equivalent in terms of long-term disease control, particularly in recurrent or advanced PSD. Off-midline flap procedures remain the most reliable option in such cases, providing durable healing through both complete disease excision and correction of the natal cleft anatomy. In summary, minimally invasive techniques should be regarded as first-line options for carefully selected primary cases, rather than universal alternatives to established reconstructive procedures. Appropriate patient selection, transparent counseling regarding expected benefits and limitations, and readiness to escalate to flap-based surgery in case of failure are essential components of a modern, evidence-based treatment strategy for pilonidal sinus disease. A proposed therapeutic decision-making algorithm is presented in Figure 7.

## 9. Prehabilitation and Adjunctive Measures

Prehabilitation and adjunctive measures should be integrated into the management pathway of PSD in a practical, patient-centered manner, as they may reduce recurrence and optimize wound healing regardless of the surgical technique adopted. Structured hair control is central. Mechanical depilation (preferably clipping rather than shaving to avoid microtrauma) can be initiated preoperatively and continued postoperatively at regular intervals (every 2–4 weeks during the healing phase). Several studies have shown that adjunctive laser hair removal is associated with a reduction in recurrence rates after surgery for pilonidal disease [68,69,70]. Laser depilation is typically initiated after complete wound healing and performed at 4–6-week intervals for multiple sessions, with maintenance treatments individualized according to hair regrowth patterns. Education on meticulous cleft hygiene is equally important. Patients should be instructed to keep the intergluteal cleft clean and dry, gently separate the buttocks during showering to remove loose hairs, and avoid prolonged moisture retention, measures consistently emphasized in long-term outcome studies and guidelines [68,69,70]. Weight management should be encouraged in overweight patients, as a deep and narrow natal cleft has been associated with higher recurrence risk; optimization of body mass index may contribute to improved local conditions and surgical outcomes [71]. Smoking cessation is strongly recommended, ideally several weeks before surgery, given its well-established association with impaired wound healing and increased postoperative complications [72]. Finally, counseling should address prolonged sitting, excessive sweating, and occlusive clothing. Encouraging regular positional changes and breathable garments may reduce maceration and hair penetration, factors implicated in disease pathogenesis and recurrence [72]. Framing these interventions as shared, modifiable risk factors empowers patients and reinforces that durable disease control depends not only on surgical technique but also on sustained behavioral measures.

## 10. Multimodal Large Language Models in Pilonidal Sinus Disease: Emerging Applications and Future Directions

The rapid expansion of artificial intelligence (AI), particularly multimodal large language models (LLMs), is beginning to influence clinical practice in dermatology and related surgical disciplines. Increasingly, patients upload images of skin lesions to AI-based platforms prior to formal medical consultation, seeking preliminary diagnostic guidance. Recent studies have demonstrated that multimodal LLMs can assist in the evaluation and diagnosis of dermatologic conditions through integrated analysis of clinical images and structured textual input, with performance approaching clinician-level assessment in selected scenarios [73]. Given that PSD is fundamentally a surface-based condition with recognizable morphological features, a similar AI-supported framework could theoretically be applied to its early identification and stratification. Standardized photographic documentation combined with structured clinical variables—such as number and location of midline pits, presence of lateral tracts, prior surgical history, body mass index, and symptom burden, could enable LLM-assisted triage and preliminary stratification toward minimally invasive or flap-based approaches. In this context, AI would function as a decision-support adjunct rather than a replacement for clinical evaluation. Looking ahead, future applications may extend beyond diagnosis to include automated cleft morphology analysis, individualized recurrence-risk prediction models, and AI-assisted shared decision-making tools that integrate anatomical, behavioral, and procedural variables. Integration with telemedicine platforms could facilitate remote follow-up, wound monitoring, and early detection of complications or recurrence through serial image analysis. However, prospective validation studies, standardized imaging protocols, regulatory oversight, and robust data governance frameworks are essential before widespread implementation. Ensuring transparency, bias mitigation, and medico-legal clarity will be critical to safe integration into routine surgical care. Overall, while AI and multimodal LLMs remain adjunctive tools at present, their evolving capabilities suggest a potential role in enhancing diagnostic accuracy, optimizing patient stratification, and supporting personalized management pathways in PSD.

## 11. Conclusions

PSD remains a common surgical condition with a substantial impact on young adults, characterized by a significant risk of recurrence and prolonged morbidity if inadequately treated. Over the past two decades, surgical management has progressively shifted away from midline excision toward off-midline closure techniques and minimally invasive approaches, reflecting increased attention to wound-related morbidity and patient-centered outcomes. Based on the strongest currently available long-term comparative evidence, including randomized trials and meta-analyses with extended follow-up, off-midline flap procedures, such as the Karydakis flap, Limberg flap, and Bascom cleft-lift, demonstrate the most consistent durability in terms of recurrence control, particularly in complex or recurrent disease. Therefore, their preferential use in these settings is supported by higher-level evidence rather than by historical convention alone. Minimally invasive techniques, including endoscopic and laser-assisted procedures, are associated with faster recovery, reduced postoperative pain, and high short-term patient satisfaction. However, most supporting data derive from observational studies and shorter follow-up periods, often in selected patients with limited primary disease. While these approaches represent valuable options in appropriately selected cases, current evidence does not yet allow for definitive conclusions regarding their long-term equivalence to flap-based surgery in advanced or recurrent PSD. Primary midline closure has consistently demonstrated less favorable outcomes in comparative analyses, particularly with respect to infection and long-term recurrence. Accordingly, its routine use appears difficult to justify considering contemporary evidence. Adjunctive strategies, including radiofrequency-assisted excision and platelet-rich plasma application, may offer potential benefits in selected contexts, but remain supported by limited and predominantly lower-level evidence. Further validation in well-designed comparative studies is required before broader recommendations can be made. Overall, optimal management of PSD should follow a patient-tailored, disease-oriented strategy that differentiates between limited primary and complex or recurrent disease, and that clearly balances short-term recovery advantages with the need for durable long-term control. Future research should prioritize high-quality comparative trials, standardized outcome definitions, and extended follow-up to strengthen the evidence base and further refine treatment algorithms.

## Figures and Tables

**Table 1:** Long-term recurrence rates according to surgical technique for sacrococcygeal pilonidal sinus disease, stratified by study design (RCTs vs. non-RCTs) and duration of follow-up, as reported by Stauffer et al. [61]. Recurrence is presented at 12, 24, 60, 120, and 240 months for primary midline closure, Karydakis and Bascom cleft lift techniques, Limberg flap, marsupialization, pit picking, and SiLaC. N.R.: not reported.

Recurrence	RCTs	Non-RCTs
Primary Midline Closure	2.1%; F.U. 12 months 7.0%; F.U. 24 months 21.9%; F.U. 60 months	3.4%; F.U. 12 months 32.0%; F.U. 120 months 67.9%; F.U. 240 months
Karydakis and Bascom cleft lift techniques	1.5%; F.U. 12 months 2.4%; F.U. 24 months 10.2%; F.U. 60 months	0.2%; F.U. 12 months 0.6%; F.U. 24 months 1.9%; F.U. 60 months
Limberg flap techniques	0.4%; F.U. 12 months 7.5%; F.U. 24 months	1.1%; F.U. 12 months 1.9%; F.U. 24 months 7.9%; F.U. 60 months
Marsupialization	1.0%; F.U. 12 months 14.3%; F.U. 24 months	1.8%; F.U. 12 months 5.6%; F.U. 24 months 9.4%; F.U. 60 months
Pit picking	4.3%; F.U. 12 months 8.3%; F.U. 24 months	2.7%; F.U. 12 months 6.5%; F.U. 24 months 15.6%; F.U. 60 months
SiLac	N.R.	1.9%; F.U. 12 months 5.1%; F.U. 24 months 36.6%; F.U. 60 months

**Table 2:** Comparative outcomes between primary midline closure and alternative closed surgical techniques (including flap procedures) based on the Cochrane meta-analysis by Al-Khamis et al. [64]. Reported outcomes include healing time (mean difference), surgical site infection (risk ratio), recurrence (Peto odds ratio, stratified by follow-up), return to work, and patient satisfaction. N.R.: not reported.

	Healing	SSI Rate	Recurrence	Return to Work	Patient Satisfaction
Primary Midline Closure vs. closed (others)	5.40 [M.D. fixed, 95% CI 2.28, 8.52]	3.72 [R.R., M-H, fixed, 95% CI 1.86–7.42]	5.76 [Peto O.R. fixed, 95% CI 2.53, 13.11] F.U. > 12 months 0.13 [Peto O.R. fixed, 95% CI 0.01, 2.26] F.U. < 12 months	N.R.	−0.90 [M.D. fixed, 95% CI −1.51, 2.26]
Limberg flap vs. closed (others)	N.R.	N.R.	7.19 [Peto O.R. fixed, 95% CI 0.44, 117.48] F.U. > 12 months	2.40 [M.D. fixed, 95% CI 2.21, 2.59]	N.R.
Karydakis techniques vs. closed (others)	N.R.	3.25 [R.R., M-H, fixed, 95% CI 1.14–9.29]	N.R.	N.R.	N.R.
Bascom techniques vs. closed (others)	9.9 [M.D. fixed, 95% CI 0.57, 170.8]	N.R.	4.5 [R.R. M-H fixed, 95% CI 0.23, 89.0]	N.R.	N.R.

**Table 3:** Clinical outcomes of Endoscopic Pilonidal Sinus Treatment (EPSiT) as reported by Emile et al. [62]. Outcomes include failure/recurrence rate, surgical site infection rate, time to return to work (mean ± SD), and patient satisfaction.

	Failure	SSI	Return to Work	Patient Satisfaction
EPSiT	6.3%	1.1%	2.9 ± 1.8 days	95.6%

**Table 4:** Comparative analysis of midline versus off-midline primary closure and flap techniques in pilonidal sinus disease according to Enriquez-Navascués et al. [63]. Outcomes include recurrence and infection risk ratios at 12 months follow-up, as well as mean difference in time to return to work between Karydakis/Bascom and Limberg procedures.

	Recurrence	Infection	Return to Work
Midline Closure vs. off-midline primary closure or flap	2.32 [R.R. 95% CI 0.98, 5.45] F.U. 12 months	2.75 [R.R. 95% CI 1.83, 4.13]	Not valuable
Karydakis and Boscom techniques vs. Limberg/modified Limberg techniques	1.12 [R.R. 95% CI 0.47, 2.63] F.U. 12 months	1.39 [R.R. 95% CI 0.61, 3.10]	−0.18 [M.D. 95% CI −0.035, −0.008]

**Table 5:** Healing outcomes and postoperative morbidity following Sinus Laser-Assisted Closure (SiLaC) as reported by Qin et al. [65]. Data include overall healing rate, healing stratified by follow-up duration (>12 months and <12 months), delayed healing, and incidence of mild postoperative pain.

	Healing	SSI
SiLAC	81.9% • 74.5%, F.U. > 12 months • 87.2%, F.U. < 12 months	11.1% (delayed healing); 18.5% mild post-operative pain

**Table 6:** Meta-analytic comparison of secondary intention healing, primary closure, and flap techniques (including Karydakis and Limberg) according to Wiinblad et al. [66]. Outcomes include odds ratios for recurrence and surgical site infection.

	Recurrence	Infection
Secondary Intention vs. Limberg techniques	0.38 [O.R. 95% CI 0.13, 1.13]	0.48 [O.R. 95% CI 0.30, 0.77]
Primary Closure vs. Flap techniques	0.31 [O.R. 95% CI 0.19, 0.51]	0.33 [O.R. 95% CI 0.23, 0.48]
Karydakis techniques vs. Limberg techniques	1.12 [O.R. 95% CI 0.66, 1.89]	2.05 [O.R. 95% CI 1.39, 3.04]

**Table 7:** Summary of surgical approaches for pilonidal sinus disease according to major international guidelines (SICCR, ASCRS, ESCP, German S3). The table outlines recommended techniques based on disease complexity, anatomical considerations, and recurrence risk, distinguishing between minimally invasive procedures (e.g., EPSiT, VAAPS, SiLaC) and flap-based off-midline excisions (e.g., Karydakis, Bascom cleft-lift, Limberg), along with their suggested indications and clinical scenarios.

Guidelines	Primary (Limited) Disease	Complex/Recurrent Disease	Midline Closure	Notes
SICCR (Italy, 2015; Consensus 2021) [14]	Minimally invasive approaches (sinusectomy, EPSiT/VAAPS) for selected primary cases.	Off-midline primary closure or flap reconstruction; secondary intention acceptable.	Discouraged.	Patient-centered approach; emphasize hair control and hygiene.
ASCRS (2019) [13]	Minimally invasive methods when expertise and equipment available.	Off-midline flap techniques (Karydakis, Limberg, Bascom) recommended.	Strongly discouraged.	Shared decision-making; consider recovery time and QoL metrics.
German S3 (2020) [15]	Pit-picking or endoscopic approaches (EPSiT/VAAPS) for uncomplicated primary PSD.	Flap-based reconstruction for complex disease; individualized approach.	Not recommended.	Structured follow-up; surgeon learning curve matters.
ESCP Consensus (2020) [16]	Supports minimally invasive strategies to reduce pain and LOS.	Off-midline closure favored when reconstruction needed.	Generally discouraged.	Calls for RCTs; limited long-term recurrence data.

## Data Availability

The data used to support the findings of this study are included within the article. The data presented in this study are available on request.
